# Copper Oxides on a Cu Sheet Substrate Made by Laser Technique

**DOI:** 10.3390/ma13173794

**Published:** 2020-08-27

**Authors:** Małgorzata Musztyfaga-Staszuk, Damian Janicki, Katarzyna Gawlińska-Nęcek, Robert Socha, Grzegorz Putynkowski, Piotr Panek

**Affiliations:** 1 Welding Department, Silesian University of Technology, Konarskiego 18A, 44-100 Gliwice, Poland; damian.janicki@polsl.pl; 2 Research and Development Center of Technology for Industry, Waryńskiego 3A, 00-645 Warszawa, Poland; ncsocha@cyf-kr.edu.pl (R.S.); office@cbrtp.pl (G.P.); 3 Institute of Metallurgy and Materials Science PAS, Reymonta 25, 30-059 Krakow, Poland; k.gawlinska@imim.pl (K.G.-N.); p.panek@imim.pl (P.P.)

**Keywords:** CuO, Cu_2_O, laser technology

## Abstract

This paper presents results from the production of copper oxide layers on a Cu sheet substrate using diode and Yb:YAG disc lasers operating in the wavelength ranges of 808–940 nm and 1030 nm. The parameters of these layers were compared with the layer obtained in the thermal process of copper oxidation at 300 °C in an infrared (IR) furnace in a natural atmosphere. Investigations into the layers mentioned above, concerning their topography, chemical composition and roughness, were made using scanning electron microscopy (SEM) and atomic force microscopy (AFM). A hot-point probe was used to determine and check the type of conductivity of the copper oxide layers formed. The optical band gap energy was estimated by applying the Kubelka–Munk method based on spectrophotometric data. Cross-sections and the element distribution maps were made using transmission electron microscopy (TEM). The phase analysis was investigated by the X-ray diffraction method (XRD). In sum, controlled laser oxidations of copper sheets allow for the formation of a mixture of Cu_2_O and CuO phases. The diode laser allows the production of a layer of copper oxides with a phase composition comparable to the oxides produced by the thermal oxidation method, while the distribution of high phase uniformity in the cross-section of the layer enables the process using a Yb:YAG disc laser.

## 1. Introduction

Due to favourable properties like low cost and abundance, metal oxide semiconductors have attracted considerable attention in photovoltaics. An example of such a compound is copper oxide, which primarily exists in either of the two well-established stoichiometric forms, Cu_2_O and CuO, corresponding to cubic and monoclinic crystal structures, respectively [1]. Both oxides are p-type semiconductors with a band gap energy (E_g_) of about 2.1 eV for Cu_2_O and 1.2–1.7 eV for CuO [2]. These two phases have mostly been analysed for photovoltaic applications. The almost optimal E_g_ for CuO makes it a very promising solar radiation harvester from the solar cell spectral dependence point of view. High optical absorption and a nontoxic nature are the significant advantages of copper oxides [3,4].

Thermal oxidation is a straightforward method of obtaining the above-mentioned oxide semiconductors. The thermal oxidation of copper is governed by by outward lattice diffusion and grain boundary diffusion. The first process favours the formation of the CuO phase and the second process favours the formation two primary mechanisms: of the Cu_2_O phase [5]. The p-type semiconductor oxides, Cu_2_O and CuO, were formed by thermal oxidation of copper sheets in the 200−1000 °C temperature range. In the range of 400−700 °C, incomplete oxidation and a mixture of Cu_2_O and CuO was obtained. Only the pure CuO was obtained at 1000 °C in an oxygen atmosphere. Carrier concentration and mobility charge carriers of CuO estimated from Hall measurements for the CuO sample were found to be 7.64 × 10^12^ cm^−3^ and 1.11 × 10^2^ cm^2^ V^−1^ s^−1^, respectively [6]. In [7], the report presented on thermal oxidation with a water vapour method gave the possibility to obtain self-supporting Cu_2_O films with high quality. The work in [8] demonstrates that a well-ordered crystalline phase of Cu_2_O is only observed above 200 °C; however, the initial oxidation of the copper films starts at about 150 °C. In the case of CuO, this phase only begins to appear above 320 °C. The oxidation behaviour of copper at a temperature below 300 °C was investigated. In [9], it was shown that the oxides that form after oxidation at low temperatures have fine crystal sizes. The grain boundary diffusion controls the oxidation process. Methods like thermal oxidation [2,5,6,7,8,9], magnetron sputtering [4] and atomic layer deposition (ALD) [10] are commonly used to manufacture copper oxides. So far, laser treatment has been used for copper sheet cleaning [11], welding [12,13] and cutting [14], along with copper oxide deposition by pulsed laser deposition (PLD) [15] or copper oxide sintering [16]. With the highest frequency, near infrared (NIR) laser has been adopted for copper welding [13], cutting [14] and other processes [16,17,18,19]. Haubold et al. proposed using a green laser for Cu welding [14], and Onyszczuk et al. proposed using this laser type for copper sheet cleaning [11]. For PLD copper oxide deposition, UV lasers are applied [15,20]. Only one paper from 1983 mentions copper sheet oxidation using an IR laser [16]. The attractiveness of laser technology arises from the possibility of precise microtreatment of surfaces due to the use of a laser beam as a noncontact, automated tool [21,22,23].

The original contribution of the presented research concerns the development of laser methods for which repeatable deposition could be easily applied for samples often used in the photovoltaic industry. In this paper, particular attention is paid to demonstrate the differences in the use of different lasers at the same stage of the oxidation process to obtain stable and repeatable working conditions for heterojunction solar cells. We present a novel, fast and relatively inexpensive approach to copper oxide production by processing the Cu sheet using simple lasers, which can easily be implemented in the industry. The research results obtained are also compared with the properties of low-temperature (300 °C) thermal copper oxide fabricated in an IR furnace. The ease with which oxidation of copper can occur is still of professional interest, as this metal-oxide system can be utilised as a material for low-cost solar cells. The results of the work confirm the potential use of the laser method to fabricate layers of Cu oxides quickly, with parameters comparable with the layers manufactured in the classic, more time-consuming and more costly annealing processes.

## 2. Experimental Details

**Figure 1:**
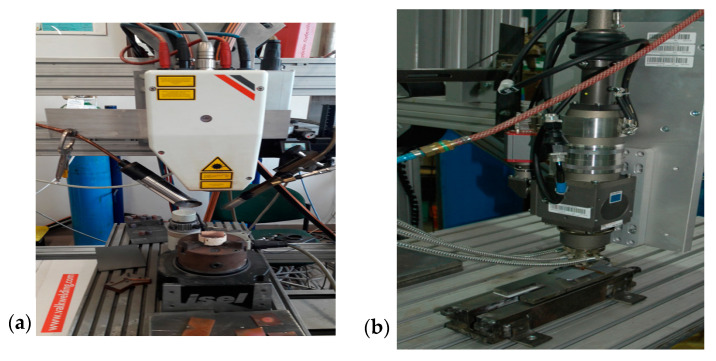
Lasers used in the experiment: (**a**) diode laser; (**b**) disc laser [24].

In the investigation, a commercial electrolytic tough pitch (ETP) R220 copper metal foil sheet with a thickness of 300 µm and a minimum size of 4 cm^2^ was applied. Two lasers were used: diode Rofin-Sinar GmbH (Hamburg, Germany) and Yb:YAG disc TruDisk Trumpf 3302 (Figure 1) (Ditzingen, Germany). Oxides were fabricated on a copper sheet using the equipment mentioned above in the natural ambient atmosphere. Table 1 presents the technical specifications of the equipment used. Table 2 presents conditions of laser treatment of the copper oxide layers on the Cu sheet substrate.

For comparison, the copper oxide fabrication process was also carried out at 300 °C in an infrared (IR) conveyer belt firing furnace for 10 min, which the samples spent in the furnace heating sectors. The IR belt furnace, made by Radiant Technology (Columbus, OH, USA), has three heating zones with tungsten lamps. Samples (marked as L3-1, L3-2 and L3-3) were chemically cleaned under the same conditions to remove industrial contaminations (e.g., oil). Next, the samples were sanded with the properly graded paper with a high index and were chemically polished just before oxidation. The cleaning solution was composed of isopropyl alcohol, HF and HNO_3_. Two samples were chosen during investigations (Table 1) based on metallographic observations of oxidised copper sheets fabricated with the laser treatment method and the available literature [25,26]. Representative samples made with the same processing time using both the diode and disk lasers were selected for a detailed analysis using transmission electron microscopy (TEM) and scanning electron microscopy (SEM) methods.

Stable conditions for the research procedure were required to obtain repeatable results. This included preparation of laser stands, preparation of samples of the same size and selection of processing parameters including warm-up time and laser beam power control, as well as the uniform heating of samples during the process.

The topography, microchemical analysis and roughness of copper oxide layers were analysed using a scanning electron microscope (SEM) with secondary electron detection with 10 kV accelerating voltage and an atomic force microscope (AFM). The optical bandwidth was determined using the Kubelka–Munk method based on spectrophotometric data. Phase formation and surface crystallisation were analysed by X-ray diffraction (XRD) using a universal powder diffractometer, the Bruker AXS Model D8 FOCUS (Cu-Kα1 radiation) (Hamburg, Germany). Research work with the TEM and STEM methods were performed for the investigation into the microstructure and used to study the phase characteristics and composition of the oxidised copper layers.

## 3. Results and Discussion

A hot-point probe was used to determine and check the type of conductivity of the copper oxide layers formed. For all tested samples, the p-type was confirmed.

**Figure 2:**
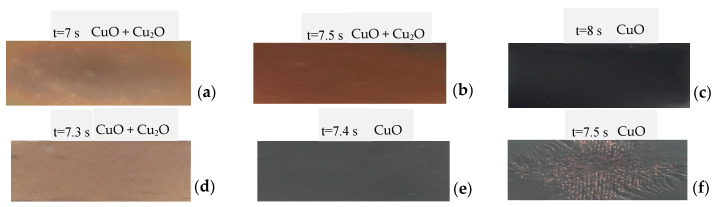
Chosen view of experimental samples after L1-2 (**a**–**c**) and L2-2 (**d**–**f**) process (where (**f**) presents an image of the sample immediately before remelting).

It was found, based on the obtained results for a diode laser, that the extended processing time is required when working parameters are set in a high-power regime. In contrast, for a low power parameter, the shorter processing time is vital; finally, the time was adjustable from 1 to 10 s. In the case of the Yb:YAG laser, the use of a high-power laser required an application of short processing time; accordingly, the application of low laser power increased the processing time significantly. The time was adjustable from 0.02 to 0.5 s. In reference to [26], the authors performed the metallographic characterisation of oxidised copper sheets. Figure 2 presents examples of the real sample images obtained with macroscopic observations after laser treatment using diode and Yb:YAG lasers.

**Figure 3:**
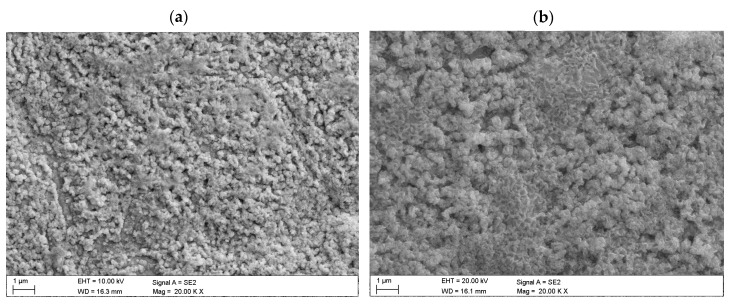
Surface topography of oxidised copper sheets (chosen example): (**a**) L1-1; (**b**) L2-4 (SEM).

The structure of the investigated copper oxide layers was determined using the scanning electron microscope (Figure 3). The contrast observed is the result of a high layer roughness occurring on all samples. The investigated samples had a porous structure with some pinholes, and their porosity depended on the laser beam power and processing time. A highly porous structure is obtained when using high laser power and long processing times. Figure 3a,b shows mixed surfaces with a very different morphology with inconsistently distributed homogeneous clusters of different size (with small dense rods).

The elemental composition of the investigated samples was analysed with the energy-dispersive X-ray spectroscopy (EDS) method. The high intensity of the copper peaks is a consequence of the considerable depth of sample penetration by the electron beam. Therefore, the signal mainly comes from the copper substrate. However, it should be noted that the ETP sheet used for the experiment contained less than 0.04% oxygen. Therefore, it certainly comes from the oxide layer on the surface, although its intensity is very low relative to the copper peak.

**Figure 4:**
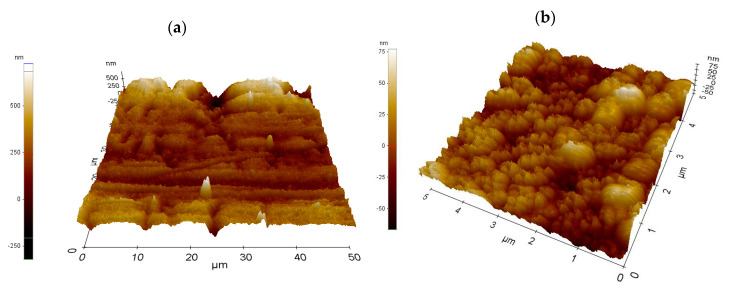
Surface topography of oxidised copper sheets: (**a**) L1-1; (**b**) L2-4.

Roughness and surface profile of the individual investigated copper oxide layers was determined using the atomic force microscope (Figure 4). In the case when the diode laser was used, the roughness of the investigated layers ranged from 0.092 to 0.331 ± 0.08 µm. In the case of the Yb:YAG laser, the roughness of the investigated layers ranged from 0.060 to 0.160 ± 0.04 µm for 4 cm^2^ samples. The energy of the Yb:YAG laser is more concentrated, and thus we can observe a more developed, highly porous structure on the L1-1 sample (Figure 4b) rather than in the case of the L2-4 sample (Figure 4a). The use of the Yb:YAG laser resulted in obtaining a more fine-grained structure than in the case of a diode laser.

**Figure 5:**
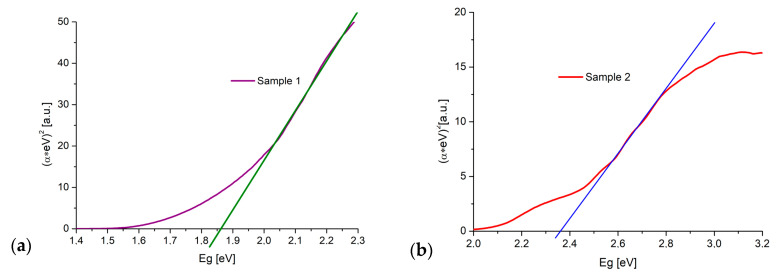
Determination of the bandgap energy using Kubelka–Munk method: (**a**) L1-1 sample; (**b**) L2-4 sample.

Figure 5 presents the results of the calculated bandgap energy obtained using the Kubelka–Munk method. The values of E_g_ = 1.85 ± 0.02 eV for L1-1 sample (Figure 5a) and E_g_ = 2.36 ± 0.05 eV for L2-4 sample (Figure 5b) suggest a mix of CuO and Cu_2_O phases in L1-1 and L2-4 samples.

**Figure 6:**
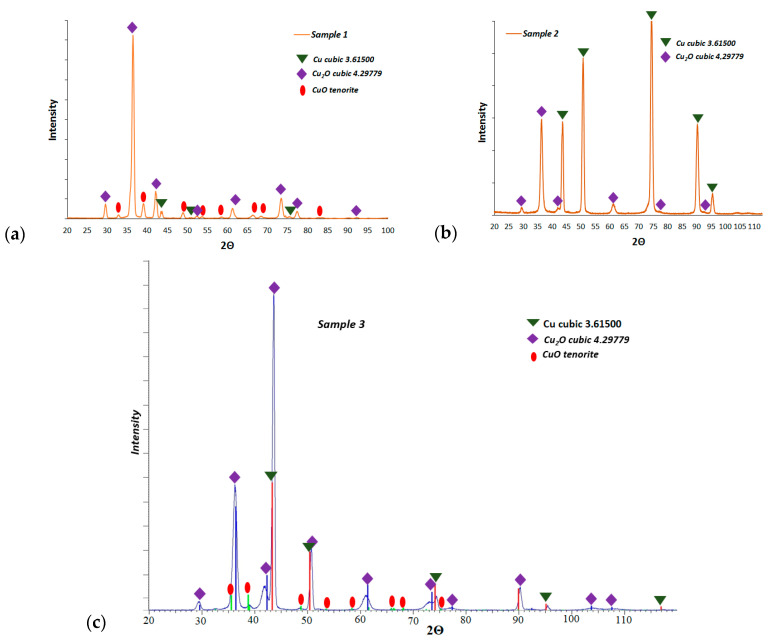
Diffraction and phase analysis results of investigated samples: (**a**) L1-1, (**b**) L2-4, and (**c**) L3-1.

Figure 6 presents the XRD results of the fabricated layers. For L1-1 and L2-4, two copper oxides were identified: CuO and cubic Cu_2_O. Figure 6c shows the XRD patterns for the L3-1 after oxidation at 300 °C. The primary phase for the surface oxidation is Cu_2_O. A smaller amount of CuO is found after prolonged oxidation at this temperature.

**Figure 7:**
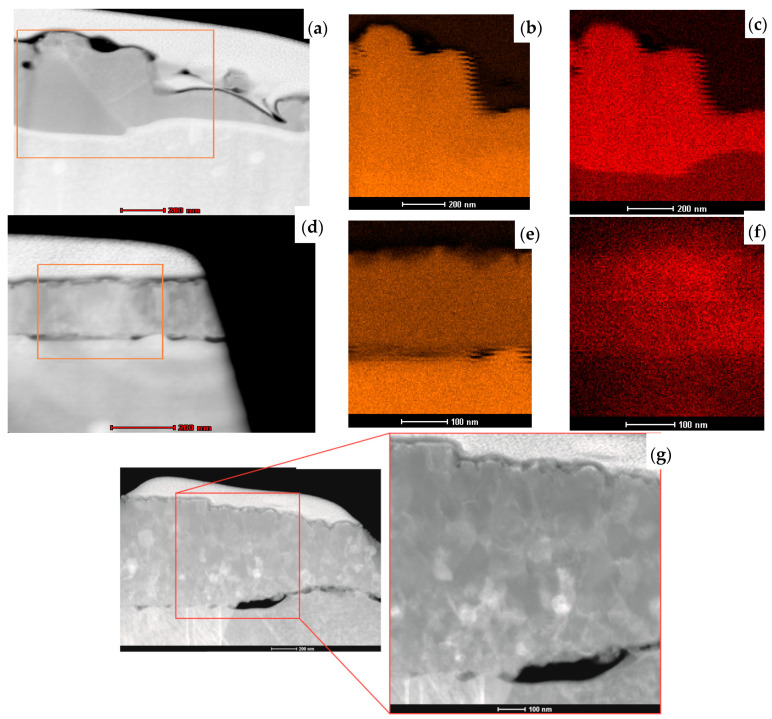
STEM cross-section images of samples (**a**) L1-1, (**d**) L2-4, and (**g**) L3-1 and element distribution maps of copper and oxygen in L1-1 (**b**,**c**) and L2-4 (**e**,**f**).

The initial microstructural analysis of the fabricated layers and the effect of the thermal process on the copper oxidation layers was conducted using scanning transmission electron microscopy. Analysis of the cross-section of the L1-1 sample (Figure 7a) revealed that the thickness of oxide produced with the 808–940 nm laser is not uniform, but no cracks on layer–substrate interface were observed. Nevertheless, some holes in the bulk layer were present. The chemical composition maps (Figure 7b,c) confirmed the homogenous distribution of copper and oxygen in the entire volume of the L1-1 sample layer. For the L2-4 sample, made using the 1080 nm laser, the cross-section proves its uniform thickness of about 200 ± 17 nm (Figure 7d). However, the obtained copper oxide layers were not concentrated well and did not adhere correctly to the Cu sheet. This may be due to the difference in the coefficients of thermal expansion (α(T)) of copper and copper oxide. Copper, with the grain size of about 10 nm, has the α(T) of 31 × 10^−6^ K^−1^, while CuO, characterised by 90 nm grains, has the α(T) of 5.1 × 10^−6^ K^−1^ [27]. The element distribution maps indicate a uniform deployment of copper and oxygen in the layer (Figure 7e,f). For the L3-1 sample, the cross-section of its ~1 μm thick layer revealed its nonuniformity (Figure 7g). Some pores can be seen between the fabricated layer and the substrate. We may state, comparing the L1-1 and L3-1 samples, that the use of a laser to create an oxide layer provided a coating that adhered well to the substrate, so this was the right direction for the investigation. For the L3-1 sample, the cross-section demonstrates its nonuniform thickness ranging from 400 nm to 1 μm (Figure 7g).

**Figure 8:**
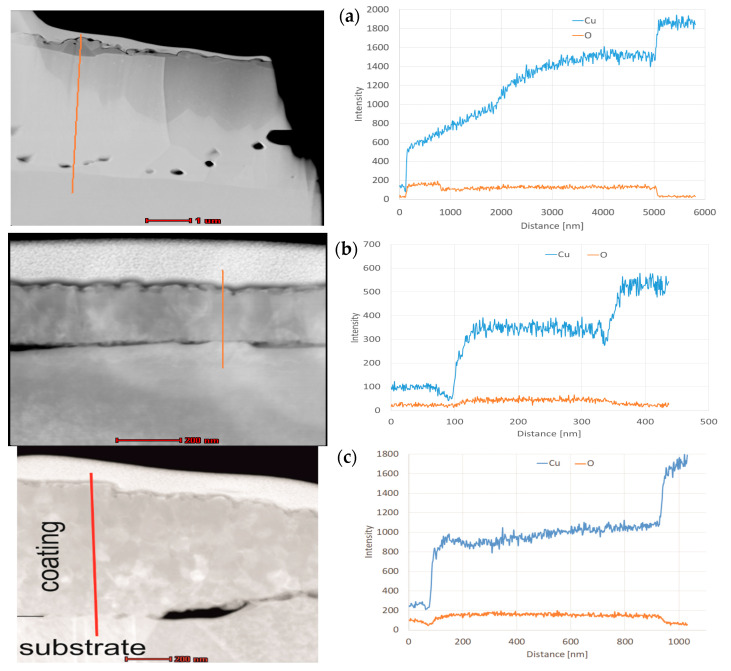
Linear change in X-ray intensity for individual elements: (**a**) L1-1 (made with diode laser), (**b**) L2-4 (made with disc laser), and (**c**) L3-1 (made with IR furnace).

The profile of chemical composition (Figure 8a,b) taken along the line perpendicular to the surface, roughly confirms that for L1-1, the amount of oxygen remains unchanged in the entire bulk at a depth from 900 to 5000 nm. In contrast, the amount of copper increases and reaches a certain plateau at a depth of 4000 nm (Figure 8b). Curve analysis suggests that the copper CuO (II) oxide is located on the surface, while copper Cu_2_O (I) oxide is situated in the middle, close to the top of the copper substrate. We also found from the cross-section that the layer thickness may even reach 5 μm. In the case of the L2-4 sample, elemental analysis indicates that the intensities of copper and oxygen are constant at a depth from 100 to 330 nm (Figure 8b). The analysis provides a 1:8 ratio of copper to oxygen intensities, which corresponds to the weight ratio of copper to oxygen in Cu_2_O. In the case of L3-1, elemental analysis indicates that the amount of oxygen remains unchanged at a depth from 100 to 950 nm, and the intensity of copper increases at a depth from 400 to 950 nm (Figure 8c).

**Figure 9:**
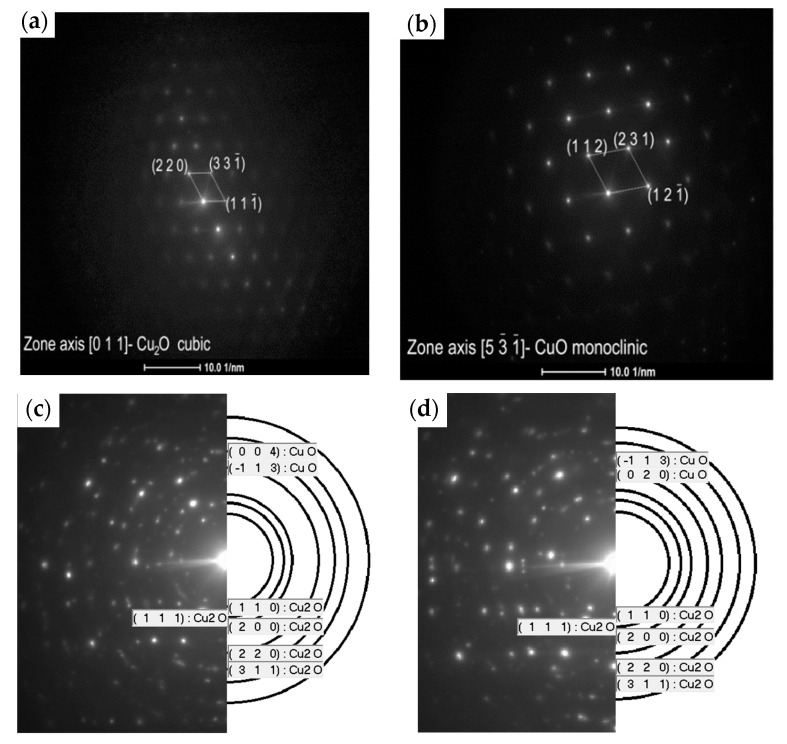
Selected area electron diffraction (SAED) patterns of the (**a**) middle layer and (**b**) top layer of the L1-1 sample and the (**c**) middle layer and (**d**) top layer of the L2-4 sample.

**Figure 10:**
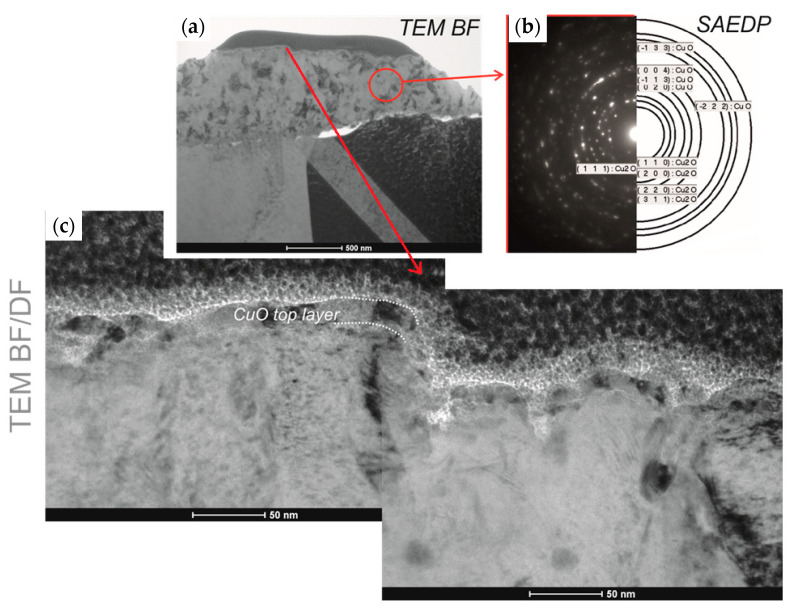
Structure (TEM) of the L3-1 sample: (**a**) bright field, (**b**) diffraction pattern from the area indicated in the bright field image, and (**c**) dark field.

Figure 9 presents the diffraction pattern (SAED) for the selected area of the fabricated copper oxides. It confirms that the L1-1 sample is well crystallised (Figure 10a,b) and is composed of the cubic Cu_2_O and monoclinic CuO, which matches the XRD pattern peaks (Figure 10a). In contrast, ring patterns for the L2-4 sample (Figure 9c,d) correspond to the highly polycrystalline material with various crystal orientations. According to [9], we can conclude that the oxidation of copper below 300 °C is also a diffusion process. The L3-1 sample is also crystallised well and is composed of the monoclinic CuO and cubic Cu_2_O structures.

## 4. Summary

The results of the material studies of copper oxides as components of heterojunction solar cells are presented in this work. The work was focused on the analysis of copper oxides fabricated with a Rofin-Sinar GmbH (Hamburg, Germany) diode laser and a TruDisk Trumpf 3302 Yb:YAG (Ditzingen, Germany) disc laser operating in different radiation ranges and the comparison of their basic material parameters with copper oxides produced in the heating process in an IR lamp furnace. All of the above processes were carried out in a natural atmosphere. The p-type of conductivity of the copper oxide layers on the investigated samples was confirmed based on the hot-point probe readings. It was found that the longest processing time of the investigated samples was 20% longer with the diode laser than with the disc laser. It was also found from SEM investigations that samples mostly showed a porous structure; their porosity depended on the parameters used, mainly the laser beam power and the processing time. The mixed phase of CuO and Cu_2_O in samples fabricated using both lasers was observed in optical investigations, mainly using the Kubelka–Munk method. The XRD results proved that phase distribution for all samples is nonhomogeneous. The quantitative distribution of the phase changes depending on the infrared wavelength of the laser used. This distribution is similar for L1-1 (processed with Rofin-Sinar GmbH (Hamburg, Germany) diode laser) and L3-1 (fabricated in the IR furnace) samples. Based on TEM results, it was confirmed that layers with a mixture of Cu_2_O and CuO were obtained for all investigated samples; however, these layers had different ratios of Cu_2_O to CuO. In the case of the L1-1 and L3-1 samples, a similarity was observed in the uneven layer thickness and obtained structure. In contrast, the thickness of the L2-4 sample was more uniform.

This paper presents a fast and relatively inexpensive method that can be easily implemented in mass production. The results of the work confirm the potential use of the laser method to obtain layers of the Cu oxides within a few seconds with parameters comparable to those of Cu oxides produced in the classic annealing processes within several minutes, which is an essential cost factor of the industrial processes.

## Figures and Tables

**Table 1:** Laser sources used.

Specifications		
	L1	L2
Manufacturer	Rofin-Sinar Laser GmbH (Hamburg, Germany)	TruDisk Trumpf 3302 (Ditzingen, Germany)
Wavelength of the laser radiation (nm)	808–940	1030
Maximum output power (W)	2000 (continuous or pulsed radiation)	3300 (continuous radiation)
Laser power range (W)	100–2000	80–3300
Focal length (mm)	82	200
Laser beam spot size (mm)	1.5 × 6.6	0.2 × 0.1

**Table 2:** Processing parameters for copper oxide layers.

Parameters						
		L1		L2		
		lb (W)		t (s)		
Ranges		500–1200		1160–1320		
**Performed Series**						
No	Symbol of series	lb (W)	t (s)	Symbol of series	lb (W)	t (s)
1	L1-1	1320	5.00–11.00	L2-1	500	9.00–11.00
2	L1-2	1160	5.50–8.00	L2-2	600	7.15–8.70
3				L2-3	700	7.15–7.40
4				L2-4	800	7.40–7.50
5				L2-5	880	6.00–7.40
6				L2-6	1200	5.00–5.85
**Chosen Samples**						
Symbol of series		Sample 1		Symbol of series	Sample 2	
		lb (W)	t (s)		lb (W)	t (s)
	L1-1	1160	7.5	L2-4	800	7.45

Designations: L1, laser 1; L2, laser 2; lb, laser beam power; t, warm-up time; L1-1, L1-2 refer to the sample number in the series made with the L1 laser; L2-1–L2-6 refer to the sample number in the series made with the L2 laser.
